# Rational Economy

**Published:** 1917-09-01

**Authors:** 


### FOOD IN WARTIME.*

### Rational Economy.

It was to be expected that the vigorous call for economy in the use of foodstuffs would be welcomed by the parsimonious as an excuse for underfeeding such persons as lay within their power. Every day brings confirmation of the fact that economy need not mean privation and is far more effectual when directed against monotony and waste than against appetites. But the administrator who is so deeply penetrated with a sense of his own services as to consider himself high above all the restrictions which common folk ought to observe is only too common in times like these, and his patriotic utterances are always of interest. A curious example of this type of mind is afforded in a controversy which excited much local interest in Yorkshire a short time ago. The North Brierley Guardians have dealt very patriotically with the inmates of their workhouse. Even in peace time the diet was somewhat monotonous, fourteen meals a week consisting of bread-and-butter, without variety of any kind. Then margarine was substituted for butter (for the able-bodied only, however); dinner consisted of one plate of food, without pudding or chance of a second helping, and twice a week "cheaper food" was substituted for meat. Such catering is too common in workhouses to call for remark. But in spite of this rationing zeal, which extended also, we understand, to the officers' diet, the Guardians maintained a rate of living at their fortnightly meetings which called forth a protest from one of themselves. Some members of the Board reside at a distance, and dinner is provided for them at a charge of 1s. 3d. a head, with tea at 6d. a head. Yorkshire teas are proverbial, but a sixpenny tea which comprises roast duck, roast beef or pork, steak pie, salads, cream, cakes, buns, and jam tarts, besides bread-and-butter, would have been esteemed a great meal even by John Brodie himself. The question which was asked was whether the Guardians would not have shown better taste, as well as a saving touch of sympathy, had they cut down their fortnightly dinners and teas so as to approximate their sacrifices to those demanded of their employees and the workhouse inmates. The discussion tailed off later into a defence of margarine for butter. There can be no real objection to the introduction of a good brand of margarine into the institutions under the Board's control. But the Guardians should ask themselves whether it is good catering to supply the inmates with a diet so intensely monotonous that a change which would be insignificant under ordinary circumstances becomes an intolerable grievance.

As a contrast to this rather one-sided kind of economy may be instanced the procedure at certain other institutions.

At Tunbridge Wells the new rations introduced for inmates of the workhouse achieve the double object of reducing the consumption of wheat and flour while satisfying those most nearly concerned. The inmates lose but two ounces of bread per head per day, and in lieu of this enjoy oatmeal and treacle for breakfast, pease-pudding, rice, or haricot beans for dinner, and cheese for tea on Sunday. The saving of bread effected is 300 lb. weekly, and the workers enjoy their lunch as usual in the form of barley-flour bread.

The Carnarvon Board adopted as new dietary a scale which allows of Irish stew for Sunday and Wednesday dinner. Pudding is allowed on two days a week; porridge is served at tea-time; and buttered oatcake for supper.

At Leeds a very interesting experiment has been made whereby the amount of bread per head per week has been reduced from 9 lb. to 4 lb.; meat has been brought down to 2½ lb., or rather less; and sugar to ½ lb. Breakfast is now a two-course meal, and consists of a pint of oatmeal with half an ounce of treacle, followed by a crisply baked 2-oz. roll of bread, ½ oz. of margarine, and ¾ pint of tea. The inclusion of porridge means that 294 lb. of oatmeal replace 1,000 lb. of bread a week, while all enjoy their meal twice as much as formerly. At Sunday dinner bread is knocked out altogether, the quantity of roast beef is lessened; 6 oz. of potatoes and other vegetables are given; and a sweet course, very popular, of rice or sago pudding is added. Other dinners are—Monday: Pint of pea-soup, 3 oz. of bread, 6 oz. of milk pudding. Tuesday: Boiled bacon, 3 oz.; haricot beans, 12 oz. Wednesday: Corned beef, an economical substitute for roast mutton, and well liked; 6 oz. of potatoes; and 6 oz. of other vegetables; with milk pudding. Thursday: Boiled bacon, with 12 oz. of pease-pudding. Friday: A meatless day; rice au gratin, 12 oz.; 2 oz. American cheese; and milk, with 2 oz. of bread. Saturday: One pint Irish stew, with 3 oz. of bread. The cost works out at about 7s. a week a head. The inmates without exception regard the new dietary as an improvement on the old, and do hard work on it with a sense of satisfaction.

The study of these and similar attempts at introducing more variety into workhouse fare suggests that much might be done in the Poor-Law institutions scattered over the country if more help as to dietaries were given by the Local Government Board. At present the precaution thought most necessary is to safeguard the quantities apportioned to each inmate. No one wants to centralise the system so that at a given moment a member of the Board could lift his hand and declare that now through the length and breadth of the country Irish stew was being consumed by the rate-supported poor. But specimen menus, differing according to locality and the time of year, with formulæ for quantities and recipes for making new dishes, would be an immense assistance in securing improved cooking and variety for the old and infirm. The mere introduction of a pudding course, to the lessening of the monstrous helping of solid meat formerly thought necessary for old people, would be a radical improvement. It requires much energy to introduce novelties into institutions well satisfied that as it was in the beginning so it should be to the end. If the energy is not found among the Guardians, might it not be set stirring from headquarters? We believe that in many localities improvements would be attempted were it possible to invoke from headquarters the aid of a practical organising food expert, preferably a woman, who could give advice as to bringing the kitchen equipment up to date, consult with the matron about difficulties, and report to the local Board of Guardians on such alterations as seemed to be expedient in the interests of the inmates and of economy.

* Previous articles appeared February 24, March 10, 24, and 31, April 14 and 21, May 5, 12, and 19, and June 9 and 16.

